# Supplementary material for: Antimicrobial and cytotoxic effects of marine sponge extracts Agelas clathrodes, Desmapsamma anchorata and Verongula rigida from a Caribbean Island
Source: PeerJ. 2022 Sep 23;10:e13955. doi: 10.7717/peerj.13955 (PMC9512013; doi:10.7717/peerj.13955)
Supplement: Supplemental Information 1 — Raw data for antibacterial assay: (A) Petri dishes photos. (B) MIC and MBC research. (C) Phot reading plate. (D) Count% viability. (E) Summury tables. [file peerj-10-13955-s001.docx]

**RAW DATA ANTI BACTERIAL ASSAY**

1. **Petri dishes Photos :**

| **Gram +** | | |
| --- | --- | --- |
| ***S. aureus*** | ***B. cereus*** | ***S. saprophyticus*** |
| 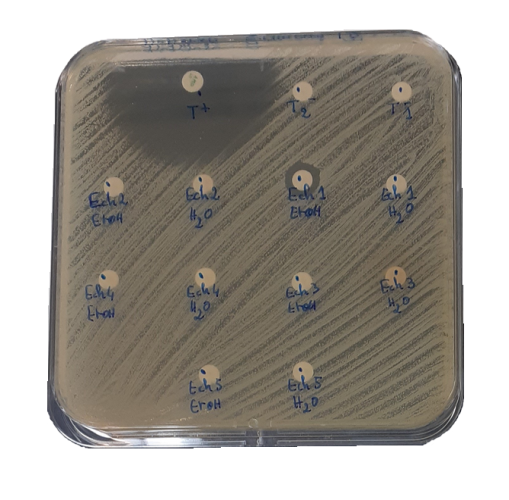 | 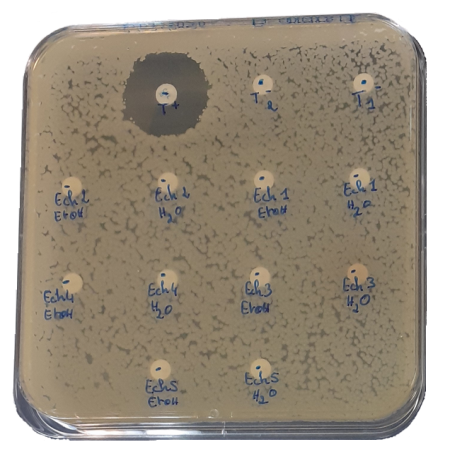 | 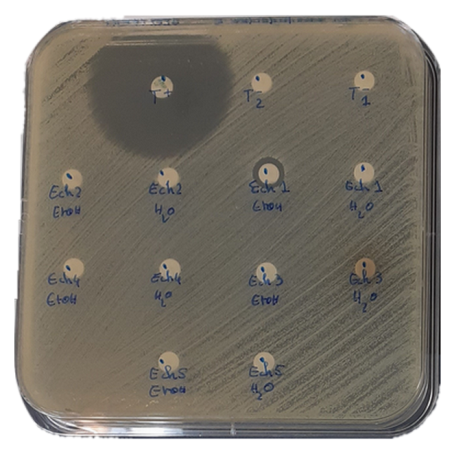 |
| 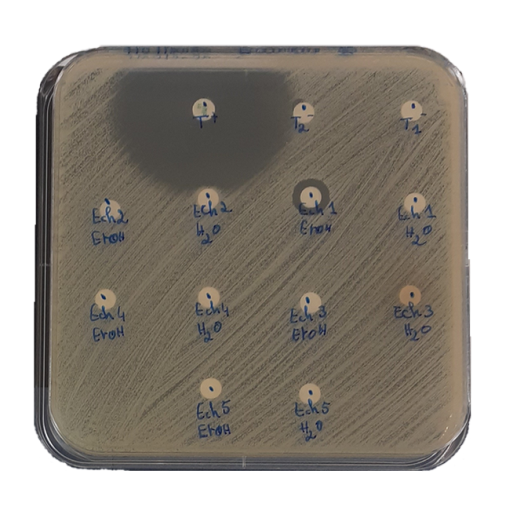 | 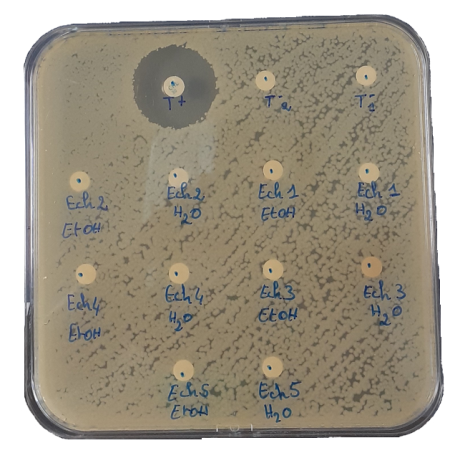 | 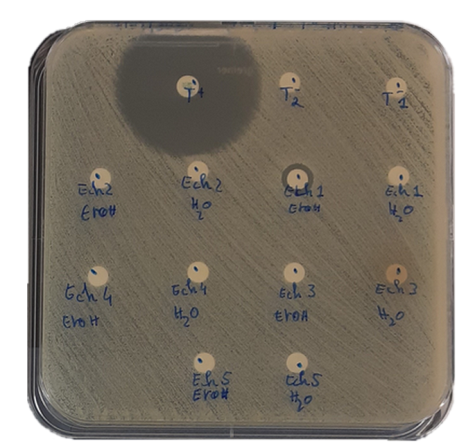 |
| 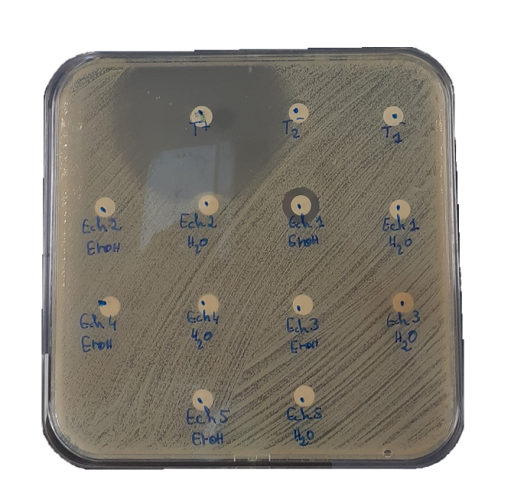 | 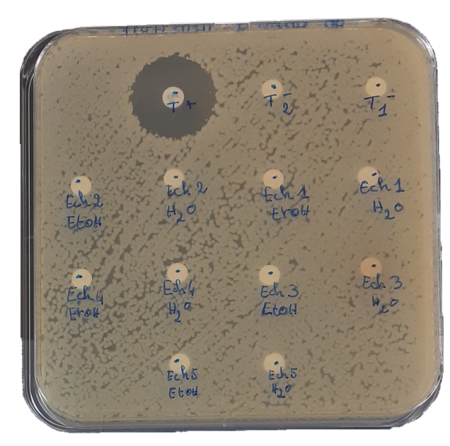 | 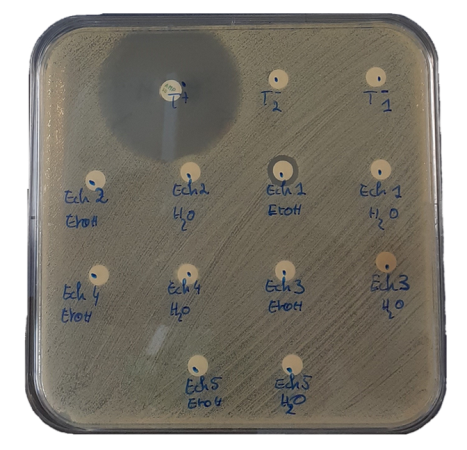 |

| **Gram +** | |
| --- | --- |
| ***E.coli*** | ***P.aeruginosa*** |
| 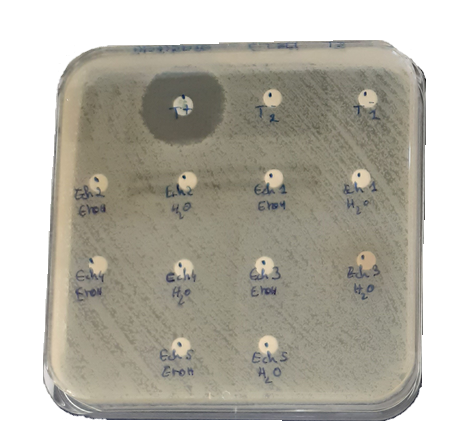 | 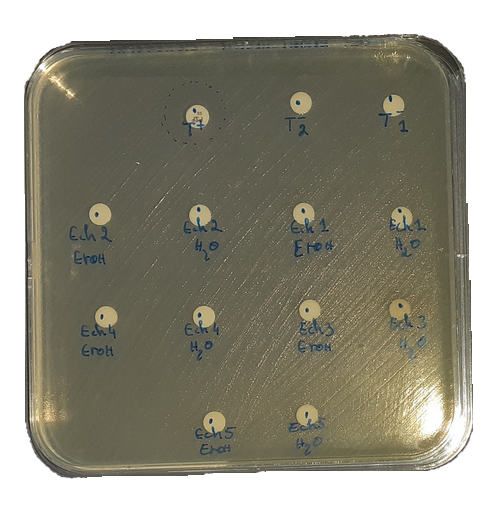 |
| 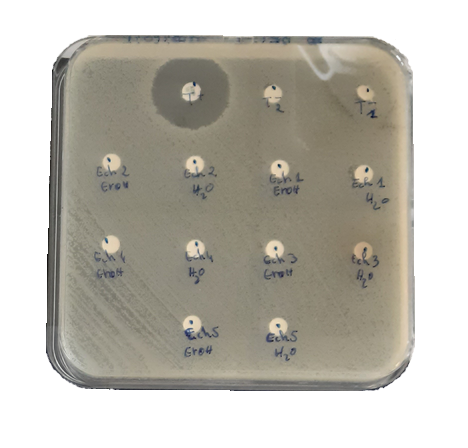 | 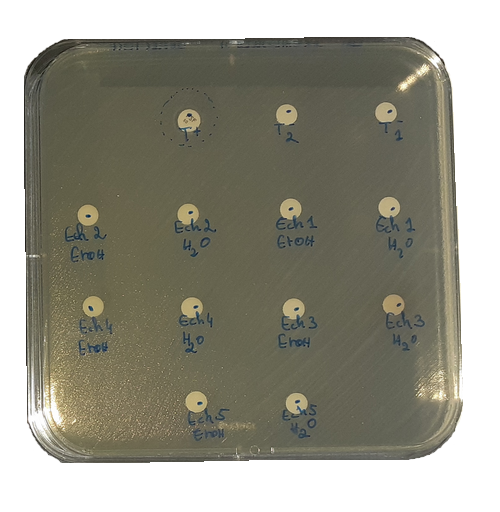 |
| 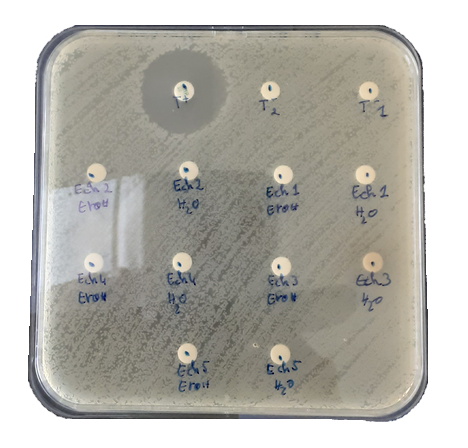 |  |

1. **MIC and MBC research :**

Minimum inhibitory concentration (MIC):

This is the lowest concentration of antibiotic (or in our case of extract) in mg/mL (or µg/mL) inhibiting any visible culture after incubation (parameters defined according to the strain).

Minimum Bactericidal Concentration (MBC):

This is the lowest concentration of antibiotic (or extract) in mg/mL (or µg/mL) leaving less than 0.01% followers of the initial inoculum (% survivors < 0.01%).

These two quantities make it possible to deduce the action of an antibiotic:

- If CMB = CMI (i.e. $\frac{CMB}{CMI}=1$) → bactericidal effect (death of bacteria),
- If CMB > CMI → bacteriostatic effect (blocking multiplication without death)

The MIC is deduced visually: it is the last tube (or well) showing no growth. The MIC value will be equal to the concentration of antibiotic (or extract) in the tube (well).

The CMB is deduced after counting the surviving bacteria of the tubes (or wells) showing no growth. It is calculated according to the formula:

$$CMB=\frac{\left[ surviving bac. \right]}{\left[ initial inoculum \right]}x 100$$

[surviving bac.] : bacterial concentration after counting the survivors in the tubes showing no growth (CFU/mL),

[initial inoculum] : bacterial concentration after counting the bacteria present in the initial inoculum (CFU/mL),

When counting on PCA only boxes containing between 30 and 300 CFU are counted. The concentration of bacteria in the inoculum (or in the wells) is calculated according to the following formula :

$$N=n x\frac{1}{V}x f$$

N: concentration of bacteria in CFU/mL

n: CFU/box between 30 and 300

V: inoculum volume in mL

f: retained dilution factor (will not be taken into account when counting survivors)

1. **Photo reading plate :**


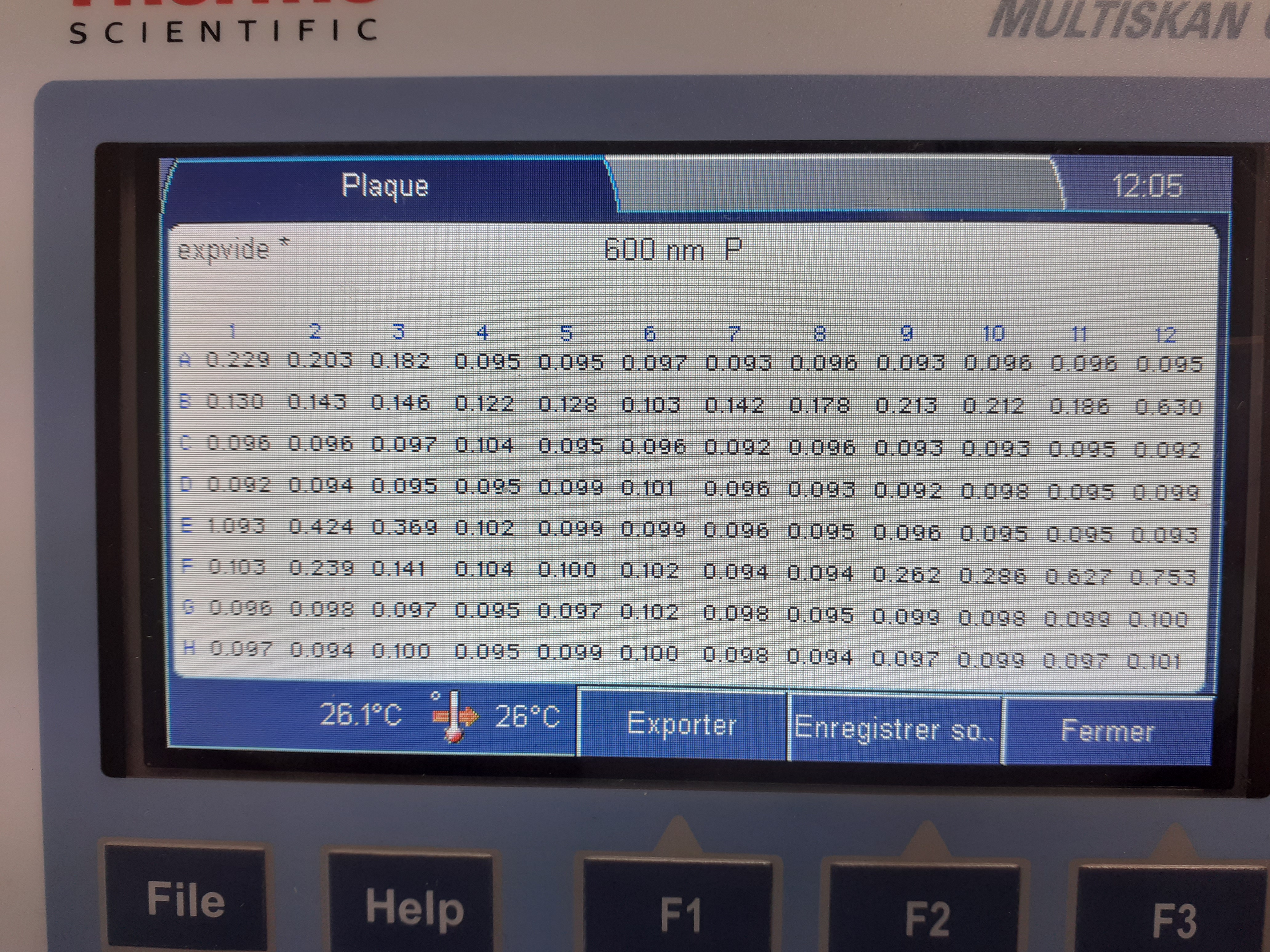


Ligne A : *Staphylococcus aureus*

Ligne B : Ech 1 EtOH

Ligne E : *Staphylococcus saprophyticus*

Ligne F : Ech 1 EtOH

1. **Count % Viability:**

| *Staphylococcus saprophyticus CIP 76125* | | *Staphylococcus aureus CIP 67.8* |
| --- | --- | --- |
| 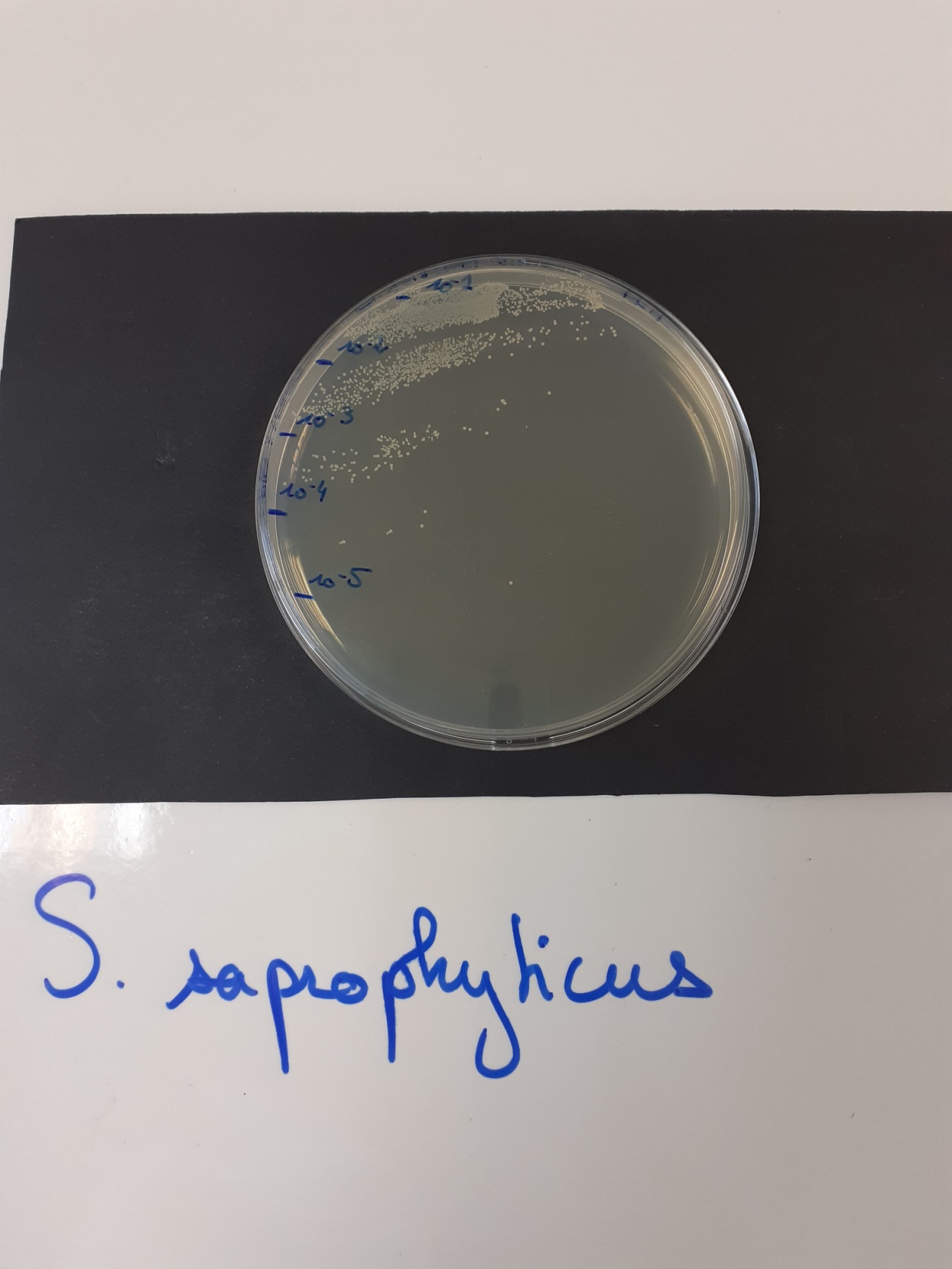 | | 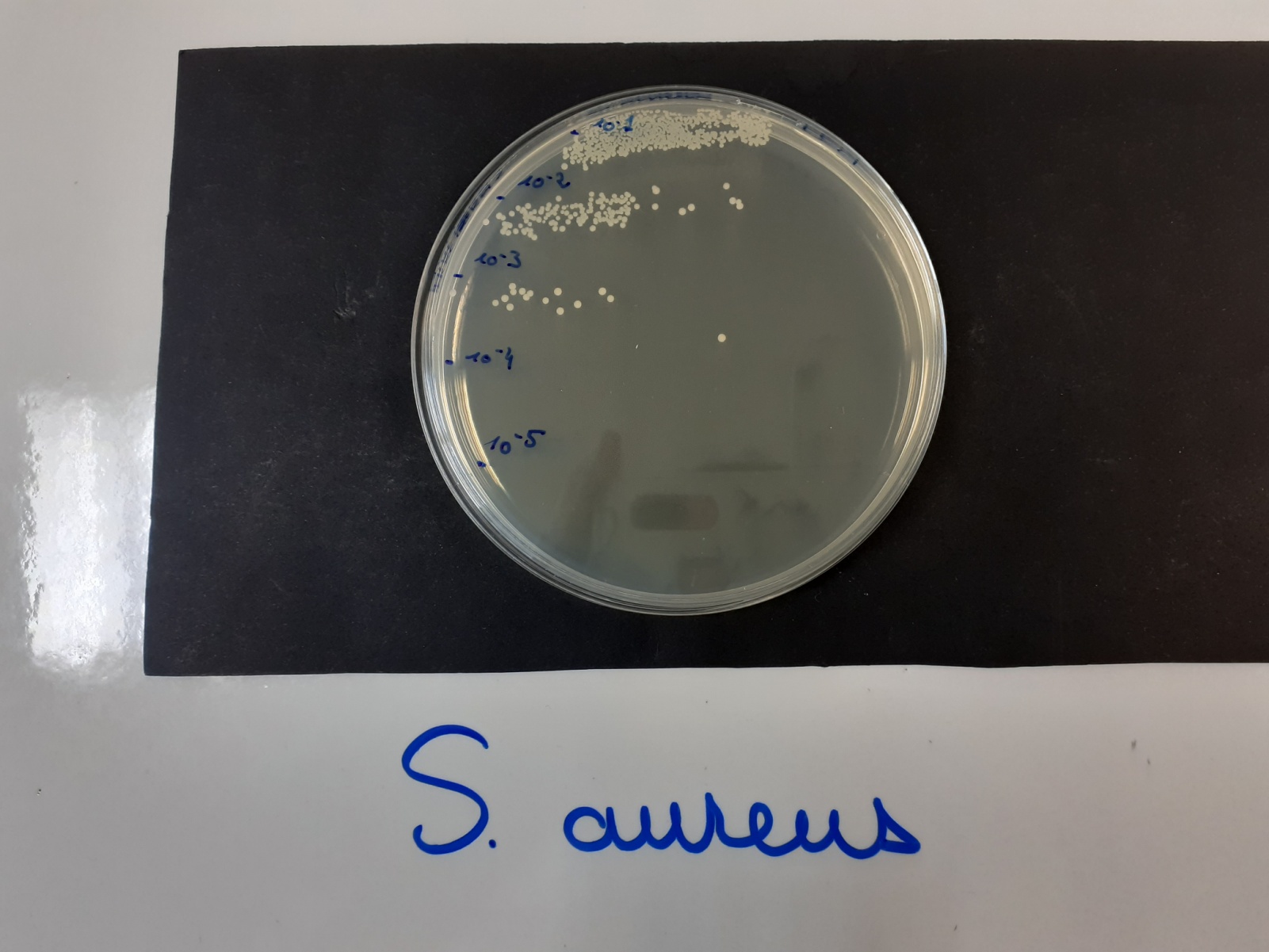 |
| Dilution | UFC | |
| 10^-1^ | NC | NC |
| 10^-2^ | NC | 115 |
| 10^-3^ | 100 | 15 |
| 10^-4^ | 6 | 1 |
| 10^-5^ | 1 | 0 |

1. **Summary Tables :**

|  | ***Staphylococcus aureus CIP 67.8*** | | | | | | | | | | | |
| --- | --- | --- | --- | --- | --- | --- | --- | --- | --- | --- | --- | --- |
| Wells | 1 | 2 | 3 | 4 | 5 | 6 | 7 | 8 | 9 | 10 | 11 | 12 |
| Dilution | 1 | 1/2 | 1/4 | 1/8 | 1/16 | 1/32 | 1/64 | 1/128 | 1/256 | 1/512 | 1/1024 | 1/2048 |
| [Ech.] µg/mL | 1000 | 500 | 250 | 125,0 | 62,500 | 31,250 | 15,625 | 7,813 | 3,906 | 1,953 | 0,977 | 0,488 |
| Trouble | - | - | - | - | - | - | - | ++ | ++ | ++ | ++ | ++ |
| D.O à 600nm | 0,13 | 0,143 | 0,146 | 0,122 | 0,128 | 0,103 | 0,142 | 0,178 | 0,213 | 0,212 | 0,186 | 0,63 |
| UFC/cupule | 0 | 0 | 0 | 0 | 0 | 0 | ± 200 |  |  |  |  |  |
| UFC/mL |  |  |  |  |  |  | 2.10^4^ |  |  |  |  |  |
| % live bac. |  |  |  |  |  |  | 1,74% |  |  |  |  |  |

- « Troubled » line : "+" trouble (bacterial growth), "-" no trouble (no bacterial growth),
- UFC : Unit Forming Colony (= number of bacteria counted on the box),
- « UFC/cupule » line : number of surviving bacteria in the wells showing no trouble,
- Grey box : analysis not performed on this well,
- « NC » : no accounting > 300 colonies,
- CMI

|  | ***Staphylococcus saprophyticus CIP 76125*** | | | | | | | | | | | |
| --- | --- | --- | --- | --- | --- | --- | --- | --- | --- | --- | --- | --- |
| Wells | 1 | 2 | 3 | 4 | 5 | 6 | 7 | 8 | 9 | 10 | 11 | 12 |
| Dilution | 1 | 1/2 | 1/4 | 1/8 | 1/16 | 1/32 | 1/64 | 1/128 | 1/256 | 1/512 | 1/1024 | 1/2048 |
| [Ech.] µg/mL | 1000 | 500 | 250 | 125,0 | 62,500 | 31,250 | 15,625 | 7,813 | 3,906 | 1,953 | 0,977 | 0,488 |
| Trouble | - | - | - | - | - | - | ++ | ++ | ++ | ++ | ++ | ++ |
| D.O à 600nm | 0,103 | 0,239 | 0,141 | 0,104 | 0,1 | 0,102 | 0,094 | 0,094 | 0,262 | 0,286 | 0,627 | 0,753 |
| UFC/cupule | 0 | 0 | 0 | 0 | 0 | 10 | 0 | 115 |  |  |  |  |
| UFC/mL |  |  |  |  |  |  |  | 1,15.10^4^ |  |  |  |  |
| % live bac. |  |  |  |  |  |  |  | 0,12% |  |  |  |  |

- « Troubled » line : "+" trouble (bacterial growth), "-" no trouble (no bacterial growth),
- UFC : Unit Forming Colony (= number of bacteria counted on the box),
- « UFC/cupule » line : number of surviving bacteria in the wells showing no trouble,
- Grey box : analysis not performed on this well,
- « NC » : no accounting > 300 colonies,
- CMI série 2
